# Effects of the PAR-1 Antagonist Vorapaxar on Platelet Activation and Coagulation Biomarkers in Patients with Stable Coronary Artery Disease

**DOI:** 10.1055/s-0039-1695710

**Published:** 2019-08-16

**Authors:** Renske H. Olie, Paola E. J. van der Meijden, Henri M. H. Spronk, Rene van Oerle, Stale Barvik, Vernon V. S. Bonarjee, Hugo ten Cate, Dennis W. T. Nilsen

**Affiliations:** 1 Laboratory for Clinical Thrombosis and Hemostasis, Cardiovascular Research Institute Maastricht (CARIM), Maastricht University, The Netherlands; 2 Thrombosis Expertise Center, Maastricht University Medical Center+ (MUMC+ ), Maastricht, The Netherlands; 3 Department of Cardiology, Stavanger University Hospital, Stavanger, Norway

On top of standard antiplatelet therapy, vorapaxar reduces the risk of ischemic events or cardiovascular death in patients with stable coronary artery disease (CAD). 1 Vorapaxar is a selective antagonist of protease-activated receptor-1 (PAR-1), thereby blocking thrombin-mediated platelet activation. 2 3 However, other actions of thrombin, such as fibrin formation and protein-C activation, are not inhibited by blocking PAR-1. Although vorapaxar probably does not affect the coagulation process directly, platelet inhibition leading to reduced availability of a procoagulant surface for the assembly of coagulation factors might lower the rate of thrombin formation indirectly. Therefore, the aim of this study is to assess whether the beneficial effect of vorapaxar on top of standard antiplatelet therapy in stable CAD patients can only be ascribed to more potent platelet inhibition or to an additional effect on thrombin generation.

To study whether vorapaxar reduces thrombin generation indirectly, we selected two upstream biomarkers of coagulation activity, factor IXa-antithrombin (FIXa-AT) and factor Xa-antithrombin (FXa-AT). As a marker of downstream coagulation activity, we measured thrombin–antithrombin (TAT) complexes. Finally, to study the additional effect on platelet activity, soluble P-selectin was assessed as a plasma biomarker of α-granule release induced by platelet activation. 4 5 6

The study was performed on plasma samples obtained from patients from three Norwegian centers participating in the Thrombin Receptor Antagonist in Secondary Prevention of Atherothrombotic Ischemic Events—Thrombolysis in Myocardial Infarction 50-trial (TRA2°P-TIMI-50). 1 The institutional review board approved the study protocol and all patients gave written informed consent. Patients with a previous history of myocardial infarction were randomly assigned to receive either vorapaxar (2.5 mg daily) or placebo in a blinded fashion, on top of standard antiplatelet agents, as managed by the treating physicians according to standards of care. 1 7 Patients using anticoagulant medication during follow-up were excluded.

A total of 135 patients with stable CAD were randomized to vorapaxar ( *n* = 73) or placebo ( *n* = 62). Baseline characteristics including age, comorbidity, and co-medication were well balanced between both groups ( Table 1 ). To study the effects of long-term treatment, blood samples were taken after a mean study drug exposure of 904 (±149) days. The use of concomitant antiplatelet agents was comparable between both groups; at the time of blood sampling, 92.6 and 22.2% were treated with aspirin and clopidogrel, respectively.

**Table 1:** Patient characteristics

	Vorapaxar ( *n* = 73) mean ± SD, or *n* (%)	Placebo ( *n* = 62) mean ± SD, or *n* (%)	*p* -Value
**Patient characteristics**			
Age (y)	61.3 ± 11.8	60.0 ± 10.3	0.48
Male	58 (79.5)	53 (85.5)	0.36
BMI (kg/m ^**2**^ )	27.6 ± 4.6	27.7 ± 3.7	0.85
eGFR (mL/min)	92.8 ± 29.1	94.1 ± 22.7	0.78
Diabetes mellitus	7 (9.6)	4 (6.5)	0.51
Hypertension	31 (42.5)	19 (30.6)	0.16
Hyperlipidemia	32 (43.8)	20 (32.3)	0.17
Treatment exposure (d)	898.5 ± 155.1	910.6 ± 143.0	0.64
**Co-medication at sampling**			
Aspirin	67 (91.8)	58 (93.5)	1.00
Clopidogrel	18 (24.7)	12 (19.4)	0.53
Dual antiplatelet therapy	17 (23.3)	11 (17.7)	0.39
**Laboratory characteristics**			
Hemoglobin (g/dL)	14.0 ± 1.1	14.3 ± 1.2	0.10
Platelet count (10 ^9^ /L)	223.9 ± 53.3	225.6 ± 57.1	0.86
MPV (fL)	10.6 ± 0.9	10.9 ± 0.8	0.18

Abbreviations: BMI, body mass index; eGFR, estimated glomerular filtration rate; MPV, mean platelet volume.

Note: Values expressed as either mean ± standard deviation (SD) or counts and percentages. Continuous variables were compared using Student's *t* -test. Categorical variables were compared using the χ ^2^ -test or Fisher's exact test when frequencies were <5.

**Fig. 1:**
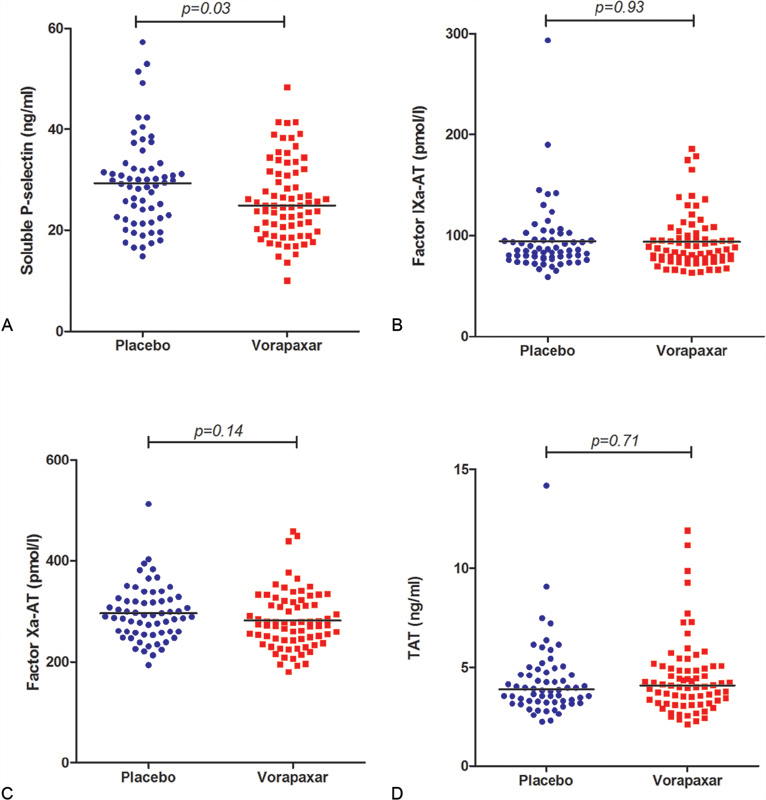
Results of soluble P-selectin, FIXa-AT, FXa-AT, and TAT complexes in vorapaxar-treated patients and placebo group. Compared with the placebo group, vorapaxar-treated patients had significantly lower levels of soluble P-selectin ( *p* = 0.03) ( **A** ). No significant differences were found in factor IXa-antithrombin (FIXa-AT) ( **B** ), factor Xa-antithrombin (FXa-AT) ( **C** ), and thrombin–antithrombin (TAT) ( **D** ) complexes between vorapaxar-treated patients and the placebo group.

According to soluble P-selectin levels, platelet activation was reduced in vorapaxar-treated patients compared with the placebo group (24.9 ng/mL [interquartile range, IQR: 20.0–31.5] vs. 29.3 [IQR: 22.3–32.5]; *p* = 0.027). No difference was found in FIXa-AT levels (94.0 ± 27.1 vs. 94.5 ± 34.0; *p* = 0.93), FXa-AT levels (282.0 ± 57.4 vs. 296.4 ± 54.6; *p* = 0.14), and TAT levels (4.0 [IQR: 3.1–5.0] vs. 3.8 [IQR: 3.0–4.9]; *p* = 0.71) between the vorapaxar-group and placebo-group ( Fig. 1 ). No differences in platelet or coagulation biomarker levels were found when comparing patients on single or dual antiplatelet therapy for both treatment groups ( Appendix A ).

**Appendix A:** Comparison of platelet and coagulation biomarkers in patients with single versus dual antiplatelet therapy

Treatment group	Biomarker	SAPT	DAPT	*p* -Value
Vorapaxar	P-selectin	26.6 ± 8.0	24.6 ± 6.8	0.36
	FIXa-AT	94.4 ± 25.5	88.4 ± 28.6	0.42
	FXa-AT	285.4 ± 62.1	265.2 ± 41.2	0.21
	TAT	4.5 ± 2.1	4.0 ± 1.3	0.41
Placebo	P-selectin	28.9 ± 8.4	31.7 ± 12.1	0.37
	FIXa-AT	96.6 ± 36.9	87.2 ± 19.3	0.42
	FXa-AT	297.4 ± 56.4	286.0 ± 47.8	0.54
	TAT	5.2 ± 2.9	3.5 ± 1.3	0.26

Abbreviations: DAPT, dual antiplatelet therapy; FIXa-AT, factor IXa-antithrombin; FXa-AT, factor Xa-antithrombin; SAPT, single antiplatelet therapy; TAT, thrombin–antithrombin.

Our data suggest that, on top of aspirin and/or clopidogrel, vorapaxar further reduces platelet activation in patients with stable CAD. This fits with the observed reduction in atherothrombotic events in these patients in the TRA2°P-TIMI-50 trial. 1 However, we found no additional effect on markers of coagulation activation, indicating that vorapaxar does not further reduce thrombin generation via intensified platelet inhibition.

Although P-selectin can also be secreted from endothelial cells, the increase in soluble P-selectin under (pre-)thrombotic conditions is assumed to be mainly derived from activated platelets. 4 Biomarkers derived from platelets have been shown to correlate with antiplatelet therapy utilization. 5 8 9 Thus, the significant reduction of soluble P-selectin levels in vorapaxar-treated patients indicates a further reduction in platelet activation on top of standard antiplatelet therapy. Our study in chronic CAD patients shows that this reduction might only occur over the course of treatment, as Storey et al have previously shown that P-selectin and sCD40-ligand were comparable between vorapaxar-treated patients and control patients in the acute phase after acute coronary syndrome. 8

Intrinsic tenase and prothrombinase complexes assemble on the procoagulant surface of activated platelets. Using our assays, the direct end products of these complexes, factor Xa and thrombin, were determined. If vorapaxar indeed reduces thrombin generation via intensified platelet inhibition, lower factor activity levels can be expected.

The finding that no difference is observed in factor IXa-AT, factor Xa-AT, and TAT complexes between the vorapaxar and placebo groups might be explained by the concomitant use of aspirin and clopidogrel, which already reduces platelet-dependent thrombin generation to such an extent that the impact of adding vorapaxar is only small. For clopidogrel, several studies have already shown an inhibitory effect on surface-generated thrombin and thrombin-induced clot formation. 10 11 Furthermore, in this study samples were taken from stable CAD patients, while the beneficial effect of vorapaxar in reducing thrombin generation might become more pronounced under conditions of acute plaque rupture, when levels of thrombin rise explosively. This is in line with the consistent reduction in the rate of type 1 (spontaneous) myocardial infarction in vorapaxar-treated patients. 1 Thrombin levels under stable conditions might be just too low to show a detectable reduction via platelet inhibition.

Finally, it is possible that vorapaxar has indeed no interference with the coagulation process, in accordance with the absence of effect on parameters of thromboelastography in a previous study. 12 Theoretically, part of the beneficial effect of vorapaxar on the risk of ischemic events might also be attributable to effects of vorapaxar on the vascular endothelium. Since PAR-1 on endothelial cells and vascular smooth muscle cells mediates mitogenic effects, vorapaxar might be effective in reducing vascular remodeling and consecutive progression of atherosclerosis.

Strengths of our analysis include the simultaneous measurement of both coagulation and platelet biomarkers in a patient population on long-term treatment with stable CAD. Limitations are the relatively small sample size, the fact that we were only able to measure at a single time point, and that total platelet P-selectin was not assessed. Although we cannot fully prove that the detected decrease in soluble P-selectin is related to additional platelet inhibition, it is likely to be at least in part a reflection of total platelet P-selectin. 4

In conclusion, our data suggest that the beneficial effect of vorapaxar on top of standard antiplatelet therapy in stable CAD patients is likely due to further attenuation of platelet reactivity, without detectable reduction in thrombin generation.
